# Ultrasound measurement of joint cartilage thickness in large and small joints in healthy children: a clinical pilot study assessing observer variability

**DOI:** 10.1186/1546-0096-5-3

**Published:** 2007-04-02

**Authors:** Anne Helene Spannow, Elisabeth Stenboeg, Mogens Pfeiffer-Jensen, Troels Herlin

**Affiliations:** 1 Department of Pediatric, Aarhus University Hospital, Section Skejby Hospital, Brendstrupgaardsvej 100, 8200 Aarhus N, Denmark; 2 Department of Pediatric, Region Hospital Randers, Skovlyvej 1, 8900 Randers, Denmark; 3 Department of Rheumatology, Aarhus University Hospital, Section Aarhus Hospital, Norrebrogade 44, 8000 Aarhus C, Denmark

## Abstract

**Background:**

Loss of joint cartilage is a feature of destructive disease in JIA. The cartilage of most joints can be visualized with ultrasonography (US). Our present study focuses on discriminant validity of US in children. We studied reproducibility between and within a skilled and a non-skilled investigator of US assessment of cartilage thickness in small and large joints in healthy children.

**Methods and results:**

In 11 healthy children (5 girls/6 boys), aged 9.6 years (9.3–10 years), 110 joints were examined. Cartilage thickness of the right and left hip, knee, ankle, 2^nd ^metacarpophalangeal (MCP), and 2^nd ^proximal interphalangeal (PIP) joint independently. The joints were examined twice, two days apart by a skilled and a non-skilled investigator. Mean cartilage thickness in the five joints was: hip 2.59 ± 0.41, knee 3.67 ± 0.64, ankle 1.08 ± 0.31, MCP 1.52 ± 0.27 and PIP 0.73 ± 0.15 mm. We found the same mean differences in CTh of 0.6 mm in the inter-observer part with regard of the PIP joint. Within investigators (intra-observer), the smallest mean difference of CTh was found in the MCP joint with -0.004 (skilled) and 0.013 mm (non-skilled).

**Conclusion:**

We found the level of agreement between observers within a 95% Confidence Interval in assessment of cartilage thickness in hip-, knee-, ankle-, MCP-, and PIP joints in healthy children. Observer variability seems not to relate to joint size but to the positioning of the joints and the transducer. These factors seem to be of major importance for reproducible US measurements. The smallest difference in measurement of cartilage thickness *between observers *was found in the PIP joint, and *within observers *in the MCP joint and it seems that using EULAR standard US guidelines is feasible for a pediatric setting. The use of US in children is promising. Studies on larger groups of children are needed to confirm the validation and variability of US in children as well as determining the smallest detectable difference of US measures.

## Background

In juvenile idiopathic arthritis (JIA), early diagnosis, initiation and optimal adjustment of aggressive therapies are essential to improve long-term outcome. This requires sensitive and specific methods for detection and monitoring the disease process. Clinical examination, laboratory tests, as well as conventional radiology are neither sensitive nor specific, in particular in the early phases of the disease.

Although destructive changes may not easily be visualized in JIA, loss of joint cartilage may be an early feature of destructive disease in JIA [1-3]. The cartilage of most joints can be visualized with ultrasonography (US). The advantages of US as a potentially useful method for frequent follow-up in pediatric patients include the method being non-invasive, easy repeatable, painless, without ionizing radiation, and relatively inexpensive.

There is increasing evidence for US being highly sensitive for early inflammatory and destructive changes in rheumatoid arthritis (RA) joints [4,5]. While systematic studies on different aspects of validation of US in RA are now emerging [6-11], documented validity assessment is needed in pediatric patients.

Our present study focuses on discriminant validity of US in children. We studied reproducibility between and within a skilled and a non-skilled investigator of US assessment of cartilage thickness in small and large joints in healthy children.

## Materials and methods

### Study design

A cross sectional point survey was utilized.

### Ethics

The parents of all participants gave informed consent. The study was conducted in accordance with the Helsinki II declaration, and was approved by the local ethical committee of Aarhus, Denmark.

### Study subjects

In 11 healthy children (5 girls/6 boys), aged 9.6 years (9.3–10.0) year*s*, 110 joints were examined.

### Ultrasonography

**Figure 1:**
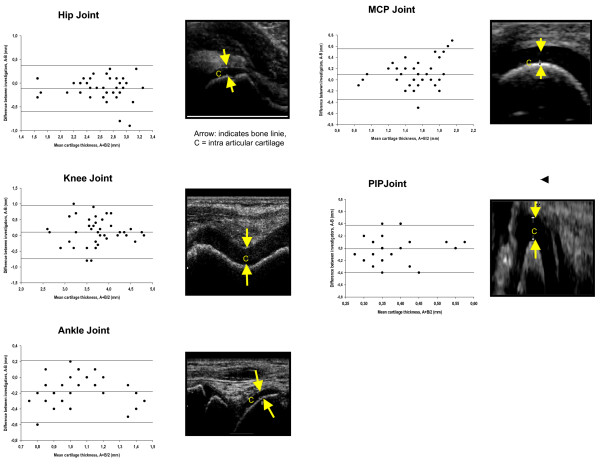
Interobserver variability.

Conventional B-mode with a linear 6–13 MHz transducer (Hitachi EUB-6500 CFM) was used. Standard scans according to EULAR guidelines were used [6]. The joints were twice examined by US in a blinded fashion. The exams occurred two days apart and were performed independently by a skilled (rheumatologist with 4 years US experience) and a non-skilled investigator (intern with 3 months US experience). Ultrasonography of the hip, knee, ankle, 2^nd ^MCP and 2^nd ^PIP joint was performed measuring the cartilage thickness (CTh) of both the right and left side respectively. The definition of CTh width of the anechoic space in the five examined joints is shown in Figure 1. In this study, we decided not to investigate synovial thickening or effusion.

### Statistical analysis

Data are given raw. Cartilage thickness was calculated as the means of all measurements. The variability within observers was calculated as: 1) The mean difference, 2) The standard deviation (SD) of the differences, along with the 95% confidence interval, and plotted by the data-differences against their mean (Bland-Altman plot) [7].

## Results

Mean cartilage thickness in the five joints was: hip 2.59 ± 0.41, knee 3.67 ± 0.64, ankle 1.08 ± 0.31, MCP 1.52 ± 0.27 and PIP 0.73 ± 0.15 mm. We found a high level of agreement between and within investigators (with a 95% CI), in assessment of CTh in hip, knee, ankle, MCP and PIP joints. (Figure 1, Table 1)

**Table 1:** Intra – observer variability between skilled and non-skilled US investigator in large and small joints.

	**HIP**	**KNEE**	**ANKLE**	**MCP**	**PIP**
**Skilled Investigator**					

Mean Differences ± SD (95% CI)^1^	0.04 ± 0.14 (-0.02 – 0.09)	-0.04 ± 0.22 (-0.13 – 0.04)	-0.23 ± 0.35 (-0.32 – 0.14)	-0.004 ± 0.14 (0.22–0.38)	0.06 ± 0.13 (0.01 – 0.12)

**Non-skilled Investigator**					

Mean Differences ± SD (95% CI)	-0.03 ± 0.18 (-0.11 – 0.04)	-0.15 ± 0.75 (-0.47 – 0.16)	-0.06 ± 0.20 (-0.14 – 0.03)	0.013 ± 0.21 (-0.01 – 0.09)	0.06 ± 0.18 (-0.01 – 0.14)

^1^Mean Differences in mm Cartilage Thickness – SD Standard deviation – 95% Confidence interval

We found the same mean differences in CTh of 0.6 mm in the inter-observer part with regard of the PIP joint. Within investigators (intra-observer), the smallest mean difference of CTh was found in the MCP joint with -0.004 (skilled) and 0.013 mm (non-skilled). Observer variability was not related to joint size.

**Figure 2:**
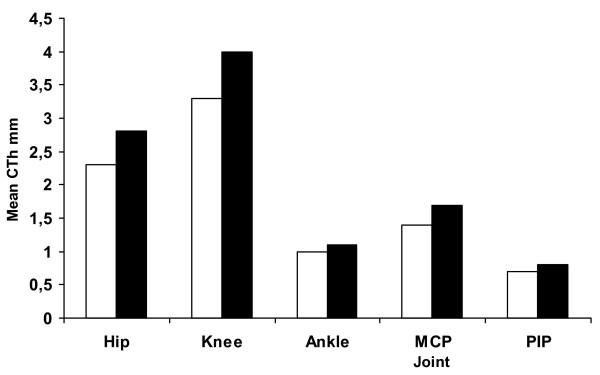
Mean Cartilage Thickness for large and small joints according to gender. Legend: □ girl; ■ boy.

We saw a clear tendency of increased mean CTh for boys in all examined joints (Fig. 2). For both genders, the greatest mean CTh was found in the weight bearing joints as the hip and knee. Because of little age variation, we cannot in this study make any assumptions about the relationship between CTh and age. This relationship could be addressed in a study of a larger population of children.

## Discussion

In this report on validation of ultrasound assessment of CTh in healthy children, we found the level of agreement between observers within a 95% CI in hip, knee, ankle, MCP, and PIP joints. Observer variability seemed not to be related to joint size, with the smallest mean difference found in measurement of cartilage thickness between observers (inter-observer) in the PIP joint, and within observers (intra-observer) in the MCP joint. With regard to the determination of bone age using US as a measurement method, a study of Castriota-Scanderbeg and colleagues [12] found similar inter- and intra-observer results when measuring CTh of the hip and knee joint (unilateral joint examinations),.

Our study is extended in the way of investigating also small joints (bilateral examination) of relevance for degenerative cartilage diseases such as JIA and to implement the EULAR US guidelines in a paediatric setting that are currently only recommended for use in adult rheumatology,. We found that the EULAR guideline is useful and that the child is capable of cooperating for the different positioning of the joints when using this US standardization.

In this present study we did not make comparative analysis between US and another imaging modality as MRI, which is believed to be the 'gold standard' in musculoskeletal imaging. A study utilizing musculoskeletal US and MRI in children would be of interest in validation of musculoskeletal US.

In our present study, but also in the study of Castriota-Scanderbeg et al, it can be concluded that positioning of joint and transducer is of major importance for reproducible US measurements. With that qualification, it appears that US measurement of cartilage thickness is a precise enough method to be used in clinical settings.

The use of US in children is promising. Studies on larger groups of children are needed to confirm the validation and variability of US in children as well as determining the smallest detectable difference of US measures.

## Conclusion

We found the level of agreement between observers within a 95% CI in assessment of cartilage thickness in hip-, knee-, ankle-, MCP-, and PIP joints in healthy children. Observer variability was not related to joint size, with the smallest difference found in measurement of cartilage thickness between observers in the PIP joint, and within observers in the MCP joint. The use of EULAR standard US guidelines appears to be feasible for a pediatric setting. The usefulness of US in children is promising and suggests the need for studies on larger groups of children that will further evaluate US of the musculoskeletal system and determine the smallest detectable difference of US measures.

## Competing interests

The author(s) declare that they have no competing interests.

## Authors' contributions

AHS carried out part of the ultrasound measurements. Performed the statistical analysis of the results, writing and coordinating the draft of the manuscript

ES carried out the study design and part of the ultrasound measurements. Drafted the manuscript

MPJ carried out the study design and ultrasound measurements. Drafted the manuscript.

TH participated in the design of the study and drafted the manuscript

All authors read and approved the final manuscript
